# Comparing the Efficacy of Temperature-Controlled Radiofrequency Tonsil Ablation versus CO_2_-Laser Cryptolysis in the Treatment of Halitosis

**Published:** 2018-05

**Authors:** Farnaz Hashemian, Hoda Jafari Moez, Mohamad Ali Seif Rabiei, Javaneh Jahanshahi

**Affiliations:** 1 *Department of Otolaryngology, School of Medicine, Hamadan University of Medical Sciences, Hamadan, Iran. *; 2 *Department of Community Medicine, School of Medicine, Hamadan University of Medical Sciences, Hamadan, Iran. *

**Keywords:** Halitosis, Laser, Pain, Tonsillitis

## Abstract

**Introduction:**

Halitosis and foreign body sensation are two common and disturbing symptoms of chronic caseous tonsillitis (CCT). The aim of this study was to compare the efficacy and safety of temperature-controlled radiofrequency (TC-RF) tonsil ablation with CO_2_-laser cryptolysis (CO_2_-LC) in the treatment of patients with halitosis caused by CCT.

**Materials and Methods:**

Sixty-two patients who suffered from halitosis and/or foreign body sensation due to CCT were enrolled in the present randomized clinical trial, and were randomly assigned into two groups. Group A underwent TC-RF tonsil ablation and Group B received CO_2_-LC. The severity of symptoms including halitosis and foreign body sensation was reported 7 days, 1 month, and 6 months after the procedure. Patient pain levels and amount of bleeding were evaluated as safety outcome measures. Pain levels were evaluated during the intervention, and at Day 1, 3, and 7 following the procedure using a visual analog scale (VAS).

**Results:**

Mean rank of pain score in the RF tonsil ablation group was found to be higher than in the CO_2_-LC group at all measured timepoints following the procedure. The amount of bleeding in the LC group was found to be significantly less than in the RF group (P<0.05). No significant difference was found between the groups regarding duration of procedure (P=0.157).

**Conclusion:**

Both procedures were found to be effective and safe in the treatment of CT-associated halitosis. However, LC showed better results based on lower pain levels, lower incidence of bleeding, and faster progression to a routine diet.

## Introduction

Palatine tonsils contain tubular crypts which extend from the tonsillar surface through the deep layers of the parenchyma. These crypts may retain keratin debris, exfoliated epithelium cells, and foreign particles, and lead to an accumulation of a yellowish soft mass termed caseum. Therefore, palatine tonsils represent an appropriate medium for active anaerobic bacteria (1-3).

Chronic caseous tonsillitis (CCT) is a common disease and has been frequently linked with halitosis (4). Indeed, it has been estimated that about 77% of patients with CCT complain of halitosis (5). 

Halitosis, an unpleasant odor present in the exhaled breath, can result in the social isolation of the patient and may eventually cause depression. 

Anaerobic proteolytic bacteria decompose organic materials and produce odoriferous compounds such as volatile sulfur, hydrogen sulfide, methyl mercaptans, and dimethylsulfide, which consequently leads to halitosis (5,6). 

CCT is characterized by caseum formation inside the tonsil crypts that may cause inflammation, leading to congestion and hypertrophy of the tonsils. Therefore, symptoms such as throat irritation and sensation of foreign bodies are common in CCT. CCT affects both men and women at any age, irrespective of their size, and can occur on one or both sides (1,2).

The initial approach for the treatment of CCT includes the use of topical antiseptics, anti-inflammatory agents, and oral antibiotics (2,7). If these treatments do not improve symptoms, surgical excision of the tonsils is indicated. However, tonsillectomy is an invasive procedure with complications such as pain and bleeding that can potentially be more serious in adults (7). 

Thus, some conservative methods such as temperature-controlled radiofrequency (TC-RF) tonsil ablation and CO_2_-laser cryptolysis (CO_2_-LC) are recommended (7-15).

This study was designed to compare the efficacy and safety of TC-RF and CO_2_-LC in the treatment of halitosis and alleviation of foreign body sensation in patients with CCT. To the best of our knowledge, the efficacy and safety of these procedures has not been evaluated in the treatment of halitosis to date.

## Materials and Methods

This randomized clinical trial was conducted at Besat Hospital, affiliated to Hamadan University of Medical Sciences, from February 2013 to September 2014. Sixty-two patients suffering from halitosis and/or foreign body sensation due to CCT who were referred to the ear, nose and throat (ENT) clinic of Besat Hospital were enrolled in the present randomized clinical trial. Sample size selection was calculated according to previous studies of TC-RF tonsil ablation and LC for the treatment of halitosis (7,14). Inclusion criteria were as follows: minimum age of 18 years; positive Finklestein’s tonsil smell test; no response to routine medical treatment (250 mg metronidazole oral tablets three times a day for 10 days and gargling of normal saline for 1 month); and normal coagulation tests. Patients with the following criteria were excluded from the trial: periodontal diseases; gingivitis; sinusitis; nasopharyngeal, oropharyngeal, or laryngeal cancer; history of other systemic diseases that result in halitosis such as hepatorenal, pulmonary, gastroenterological diseases (such as gastroesophageal reflux), or diabetes mellitus. Moreover, patients who had contraindications to RF treatment of the tonsils (e.g. having a cardiac pace maker, immunodeficiency diseases, or coagulation disorders) and those who had limitations with regards to CO_2_-LC (e.g. anatomical oropharyngeal anomalies, Mallampati grading 3 or 4, cleft palate, limitations in terms of opening the mouth, and other oropharyngeal abnormalities) were excluded from the study.

The present study was approved by the ethics committee of Hamadan University of Medical Sciences and all participating patients were informed of the study procedure, and signed written consent forms. Moreover, the study protocol was registered in the Iranian Registry of Clinical Trials(ID: IRCT201303233186N3).

The origin of halitosis was established by Finkelstein’s tonsil smelling test which is based on massaging the tonsils and smelling the squeezed discharge. The test was performed by the examiner who pressed the anterior pillar with a tongue depressor in order to obtain caseum, and allowed the patient and his or her close family member to smell the resulting odor. If the fetid odor of caseum reflected the typical malodor of the patient, the test was considered positive. In patients who had foreign body sensation, if removal of the caseum led to improvement in the symptom, diagnosis was established. The severity of halitosis and foreign body sensation was evaluated preoperatively using a visual analog scale (VAS) such that a score of 0 represented no related symptoms while a score of 10 indicated the worst experience of related symptoms. Patients were randomly assigned to two groups using balanced block randomization. For this purpose, four sheets of paper were prepared. The letter “R” meaning “radiofrequency” was written on two sheets and the letter “L” meaning “Laser” was written on the other two sheets. The paper sheets were then placed in a container. An independent third party randomly took one paper at a time from the container for each patient without replacing the paper until the four sheets were removed. After that, all paper sheets were placed back into the container and the action was repeated several times to reach the required sample size. 

*Surgical technique*

Both procedures were performed under local anesthesia with lidocaine spray and an injection of lidocaine 2% at the anterior and posterior pillar. All caseum from both tonsils was removed by applying pressure on tonsils.

TC-RF tonsil ablation was achieved with the insertion of an RF probe into the tonsillar crypts at 10 W power (Celon lab ENT, Model: REF LAB 006.025.001) (200-970 V). CO_2_-LC was performed using an A.R.C LASER (GmbH model C-Las Type) with a nominal power of 30 W, attached to an articulated arm handpiece, with applications of 10 W continuous wave laser power, with a focus point of 2 mm. The handpiece did not touch the tonsils. Protective glasses were used by the patients and the medical team. According to the Brodsky scale, grade 1 and 2 tonsils received RF or laser beam in 4 points, and grade 3 and 4 tonsils received the same in 6 points (16). All patients were kept under observation in the hospital for 4 hours following the procedure, and were discharged with the recommendation to eat soft foods, gargle normal saline two times a day, and take 325 mg acetaminophen every 6 hours if required.

Patient pain levels and amount of bleeding were evaluated as safety outcome measures. Pain level was evaluated during the intervention, and at Day 1, 3, and 7 following the procedure using a VAS. Patients were also asked after how many days following the procedure they were able to return to a regular diet and were able to go back to work.

The amount of bleeding during the procedure was recorded using the following grading: Grade 0, no bleeding; Grade 1, some oozing requiring no intervention; Grade 2, minimal bleeding which could be controlled by compression; Grade 3, severe hemorrhage requiring surgical intervention. Patients were reexamined 7 days, 1 month, and 6 months following the procedure, and the severity of halitosis and foreign body sensation was recorded postoperatively using the following VAS: Score 0, no related symptoms; Score 10, worst experience of related symptoms. The amount of improvement was measured as follows: Improvement, >30% decrease in severity of symptoms; No improvement, <29% decrease in severity of symptoms. Data obtained were analyzed using SPSS software (version 16.0, SPSS Inc, Chicago, Illinois). Independent sample t-tests and the Mann-Whitney test were used to compare the efficacy of the procedures. Moreover, Chi-square tests were used for qualitative analyses if related. P-values less than 0.05 were assumed to be statistically significant.

## Results

**Fig 1:**
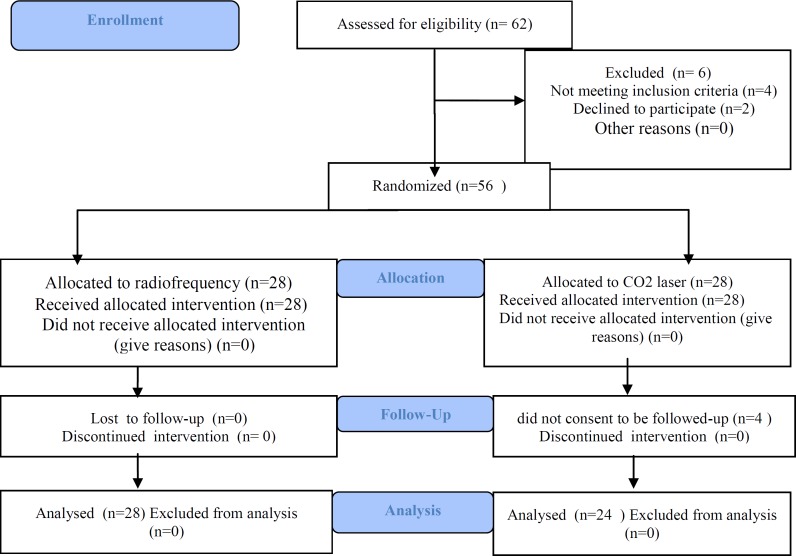
Enrollment and Follow-up Procedure of the Patients

The study included 56 of the 62 patients initially enrolled. Twenty-eight patients underwent TC-RF tonsillar ablation (Group A) and 28 patients underwent tonsillar ablation with CO_2_-LC (Group B). Four patients were not available for follow-up (Fig.1).

The proportion of male patients in Group A and Group B was 42.9% and 25%, respectively. The mean age ± standard deviation (SD) was found to be 27.8±8.1 years in Group A and 26.2±6.3 years in Group B. No significant differences were found between the two groups regarding age, gender, foreign body sensation, and halitosis before the study (P>0.05). Demographic characteristics of the patients are shown in (Table.1).

**Table 1:** Demographic and Baseline Characteristic of two Groups

	**Group A** 1 ** (n=28)**	**Group B** 2 ** (n=24)**	**P-Value**
Male	12 (43%)	6(25%)	0.44
Female	16 (57%)	18 (75%)	
Age mean (SD)	27.8 (8.1)	26.2 (6.3)	0.17
Foreign body sensation*	1.89	3.58	0.081
Halitosis *	5.35	3.54	0.100

* According to VAS mean range (between0-10)

1 Group A: Temperature-Controlled Radiofrequency Tonsillar Ablation

2 Group B: Co_2_- Laser Cryptolysis

Degrees of improvement of halitosis and foreign body sensation in each group are shown in (Table 2,3), respectively. Seven days following the procedure, 68.4% of patients in the TC-RF group and 84.6% of the patients in CO_2_-LC group showed improvements in halitosis. Both procedures significantly improved the symptoms of halitosis and foreign body sensation (P<0.05). Moreover, significant differences were found between the groups in terms of improvement of halitosis at Month 1 (P=0.003) and Month 6 (P=0.014) following the procedure.Mean halitosis and foreign body sensation in the two groups before and at different time points after the intervention are shown in( Table.4).

Mean rank of pain score of both groups (according to VAS) during intervention, at Day 1, 3, and 7 following the procedure are shown in (Table 5). Pain levels in Group A were greater than in Group B at all time points, and the differences between the two groups were statistically significant (P<0.05).

**Table 2:** Degree of Halitosis Improvement at Different Time Points Following Interventions in Both groups

	**Group A** 1 ** (n=19)**	**Group B** 2 ** (n=13)**	**p-Value**
Day 7			
Improved, n (%)	13 (68.4)	11 (84.6)	0.192
Not improved, n (%)	6 (31.6)	2 (15.4)	
Month 1			
Improved, n (%)	16 (84.2%)	10 (76.9%)	0.003
Not improved, n (%)	3 (15.8%)	3 (23.1%)	
Month 6			
Improved, n (%)	15 (78.9%)	10 (76.9%)	0.014
Not improved	4 (21.1%)	3 (23.1%)	

1 Group A: Temperature-controlled radiofrequency tonsillar ablation

2 Group B: CO_2_-laser cryptolysis

**Table 3:** Frequency of foreign body sensation at different time points following interventions in both groups

	**Group A** 1 ** (n=9)**	**Group B** 2 ** (n=11)**	**p-Value**
Day 7			
Improved, n (%)	7 (77.8)	9 (81.81)	0.028
Not improved, n (%)	2 (22.2)	2 (18.19)	
Month 1			
Improved, n (%)	8 (88.9)	10 (90.9)	0.111
Not improved, n (%)	1 (11.1)	1 (9.1)	
Month 6			
Improved, n (%)	7 (77.8)	10 (90.9)	0.028
Not improved, n (%)	2 (22.2)	1 (9.1)	

1 Group A: Temperature-controlled radiofrequency tonsillar ablation

2 Group B: CO_2_-laser cryptolysis

**Table 4:** Mean of halitosis and foreign body sensation before and after the interventions in the two groups at different time points according to VAS

	**Group A** 1		**Group B** 2	
	**Halitosis**	**Foreign body**	**Halitosis**	**Foreign body**
Before, mean (SD)	5.35 (4.2)	5.88 (1.45)	3.54 (3.7)	7.16 (1.99)
Day 7, mean (SD)	2.53 (3.2)	2.77 (2.48)	0.79 (1.8)	3.33 (3.02)
Month 1, mean (SD)	2.57 (2.9)	2.44 (2.13)	1.04 (1.89)	3.5 (2.19)
Month 6, mean (SD)	3.17 (3.69)	2.66 (2.12)	1.12 (1.94)	4.1 (3.01)

1 Group A: Temperature-controlled radiofrequency tonsillar ablation

2 Group B: CO_2_-laser cryptolysis

**Table 5:** Mean of halitosis and foreign body sensation before and after the interventions in two groups at different time points according to VAS

	**During intervention**	**Day 1**	**Day 3**	**Day 7**
Group A^1^, mean	33.96	31.48	31.00	32.18
Group B^2^, mean	17.79	20.69	21.25	19.88
p-value, mean	<0.001	0.010	0.018	0.001

1 Group A: Temperature-controlled radiofrequency tonsillar ablation

2 Group B: CO_2_-laser cryptolysis

Six patients (21%) in the TC-RF group were reported to have oozing that stopped spontaneously. No bleeding was reported in the other patients. In the CO_2_-LC group, only one patient (4.1%) had oozing, which was significantly less than in the TC-RF group (P<0.05). There was no statistically significant difference between the groups regarding duration of procedure (P=0.157); however, the speed with which a regular diet was resumed was faster in Group B than in Group A. Patients had moved to a regular diet after 3.1 days in Group A and after 1.9 days in Group B (Table.6)

**Table 6:** Comparison of two groups regarding beginning of regular diet and duration of procedure

	**Group A** 1 ** (n=28)**	**Group B** 2 ** (n=24)**	**p-Value**
Return to regular diet (day), mean (SD)	3.14 (2.32)	1.91 (1.05)	0.021
Duration of procedure (min), mean (SD)	6.41 (1.99)	6.10 (3.03)	0.157

1 Group A: Temperature-controlled radiofrequency tonsillar ablation

2 Group B: CO_2_-laser cryptolysis

A statistically significant difference was found between the groups regarding the amount of time required to return to routine work following the procedure (P=0.052).

No adverse effects or complications were encountered during the study.

## Discussion

CCT is characterized by caseum retention and related symptoms such as halitosis and foreign body sensation. Failure of clinical treatment indicates tonsillectomy, which is likely to have side effects including, in particular, hemorrhage and pain. Recently, conservative procedures such as CO_2_-LC and TC-RF tonsillar ablation have been introduced. In these procedures, the crypt ostium is opened, thus avoiding caseum retention. In the present study, the efficacy and safety of the mentioned methods in the treatment of halitosis and alleviation of foreign body sensation in patients with CCT were compared. To the best of our knowledge, the efficacy and safety of these procedures has not previously been evaluation in the treatment of halitosis.

According to the results of our study, halitosis and foreign body sensation were significantly reduced following both procedures. Pain level during the intervention and at all time points following the procedure was higher in the group who underwent TC-RF compared with CO_2_-LC.

Dal Rio et al. previously evaluated the effects of CO_2_-LC in 38 patients with halitosis. According to their results, all patients showed improvement in halitosis after the procedure, which is consistent with the results of the present study. Moreover, volatile sulfur compounds were found to be decreased by 30.1%. Caseum retention was also significantly decreased (13).

Finkelstein et al. studied the efficacy of CO_2_-LC in the treatment of halitosis in 53 patients with CCT. Complete elimination of halitosis was reported in 52.8% of the patients with a single session of CO_2_-LC. Two sessions were required in 34% of patients for complete elimination of halitosis, while three sessions were needed in 9% of patients. Overall, resolution of halitosis was reported in 92% of patients. In our study, 6 months following the procedure, patients who underwent CO_2_-LC showed improvement in halitosis and foreign body sensation by 76.9% and 90.9%, respectively. Consistent with results of the present study, Finkelstein et al. concluded that CO_2_-LC is an effective, safe, and well-tolerated procedure for the treatment of halitosis in patients with CCT (7).

In a retrospective study, Tanyeri et al. evaluated the efficacy and safety of TC-RF tonsil ablation in the treatment of halitosis caused by CCT in 58 patients. complete elimination of halitosis was reported with one session in 84.4% patients and after two sessions in 6.9% patients,totally 91.3% had complete disappearance of halitosis. These findings support the results of our study. In our study, 6 months following the procedure, patients who underwent TC-RF tonsil ablation showed improvement of halitosis and foreign body sensation by 78.9% and 77.8%, respectively. Moreover, similar to the results of our study, the authors concluded that TC-RF tonsil ablation is effective in the treatment of halitosis caused by CCT (14).

In another retrospective study, effectiveness of RF cryptolysis in the treatment of chronic CCT-related halitosis was investigated. According to the results, mean VAS score was significantly reduced from 6.82±1.45 at the beginning of the study to 0.88±2.5 after 12 months. Moreover, following one session of RF cryptolysis, 76.5% of patients were found to be negative in Finkelstein's test (8).

In a prospective trial, use of a bipolar electrode to administer RF cryptolysis was compared with use of a monopolar electrode in the treatment of patients with halitosis. Better results were found with bipolar RF cryptolysis in comparison with the monopolar case. Thus, bipolar RF cryptolysis was recommended for the treatment of halitosis and tonsillitis (15).

In our study, the level of pain was found to be greater in the RF group than in the LC group. This is probably due to the possible damage done to the neurovascular system and muscles in the tonsillar bed.

In our study, 21% of patients in the TC-RF group were reported to have oozing that stopped spontaneously. No bleeding was reported in other patients. Among patients who underwent LC, only one (4.1%) had oozing, which was at a significantly lower rate than in the TC-RF group (P<0.05). In the study by Tanyeri et al., only one out of 58 patients experienced a self-limited bleeding during the RF procedure. Additionally, one patient experienced bleeding 24 hours following the procedure and underwent tonsillectomy. Our findings are consistent with the results of previous studies and confirm that there is a minimal risk of bleeding during and after both TC-RF tonsil ablation and CO_2_-LC. Indeed, this is a great advantage of these procedures over tonsillectomy in the treatment of CCT-associated halitosis. However, it should be noted that in patients with a strong gag reflex, the most feasible treatment is known to be tonsillectomy.

The difference between the two groups regarding advancement to a regular diet was statistically significant (3.1 days for RF versus 1.9 days for LC). In the study by Finkelstein et al., patients returned to a regular diet 1 to 3 days following LC, which is similar to the results of the present study (7).

According to Karadag et al., RF ablation results in changes in the microbiology of the tonsils. These researchers found a statistically significant reduction in bacterial number following RF administration in comparison with the control group (10). Therefore, one may conclude that RF ablation probably leads to a reduction of halitosis for a short time. We followed the patients for 6 months, and no significant difference was found in the level of halitosis at Week 1 and Month 1 after the procedure. These findings have also been demonstrated by previous studies in which patients were followed up over a longer time period.

According to our results, both procedures were found to be effective in the treatment of CCT-associated halitosis in adult patients. However, LC was found to be a better choice based on a lower level of pain and bleeding and faster progression to a routine diet. It should be noted, however, that LC costs much more than RF ablation, and is not indicated in all patients. For instance, patients with a strong gag reflex or anatomical abnormalities of the mouth or throat, and patients who have a reduced mouth opening are not good candidates for LC and might be better treated with RF ablation.

## Conclusion

TC-RF tonsil ablation and CO_2_-LC were both found to be effective and safe procedures for the treatment of CCT-associated halitosis, while avoiding the post-procedure morbidities and discomfort of tonsillectomy. However, LC probably has advantages over RF ablation due to lower levels of post-operative pain and faster progression to a routine diet.
